# Implicações de Custo e Perspectivas de TAVI versus SAVR no Ambiente Hospitalar

**DOI:** 10.36660/abc.20250669

**Published:** 2025-11-27

**Authors:** Renato Paladino Nemoto, Roney Orismar Sampaio

**Affiliations:** 1 Hospital das Clínicas da Faculdade de Medicina da Universidade de São Paulo Instituto do Coração São Paulo SP Brasil Instituto do Coração do Hospital das Clínicas da Faculdade de Medicina da Universidade de São Paulo, São Paulo, SP - Brasil

**Keywords:** Custos e Análise de Custo, Implante Transcateter de Valva Aórtica, Substituição da Valva Aórtica Transcateter, Terapêutica

O artigo " Implante de Valva Aórtica Transcateter vs. Cirurgia de Valva Aórtica em um Hospital Brasileiro do Sistema Único de Saúde (SUS): Resultados e Custos Periprocedimentais"^1^ aborda um tema relevante, especialmente após a inclusão do implante transcateter de válvula aórtica (TAVI) no rol de procedimentos disponíveis no SUS. Pacientes submetidos ao TAVI são tipicamente mais velhos, com mais comorbidades e mais frágeis. Por outro lado, a substituição cirúrgica da valva aórtica (SAVR) em pacientes idosos pode resultar em aumento da morbidade e do tempo de internação hospitalar.^2^

No estudo, o risco cirúrgico foi baixo em ambos os grupos (STS 3,62±2,28 no grupo TAVI vs. 1,64±0,94 no SAVR, p<0,001), o que pode demonstrar a limitação da estratificação de risco cirúrgico com as ferramentas atuais.^3^ A mortalidade foi menor no grupo TAVI (2,8% vs. 5,0%), mas sem atingir significância estatística. A estratégia minimamente invasiva (sem anestesia geral, com monitorização por ecocardiografia transtorácica) resultou em menor tempo de internação hospitalar (2,0 [1,0; 4,0] dias no grupo TAVI vs. 8,0 [7,0; 12,0] dias no grupo SAVR, p<0,001). No entanto, os custos foram maiores que os do SAVR. Os altos preços dos materiais (R$ 55.750,90 [52.345,30; 92.286,8] TAVI vs. R$ 22.518,10 [19.130,60; 25.875,10] SAVR, p<0,001) são um fator significativo. Complicações tardias, como maior taxa de infecção hospitalar, incluindo infecção de ferida operatória, são desfavoráveis à cirurgia, devido ao aumento de internação para uso de antimicrobianos, recursos humanos hospitalares, entre outros custos.

As diretrizes nacionais e internacionais atuais indicam uma preferência por TAVI em pacientes com alto risco cirúrgico ou com fragilidade significativa em relação à SAVR.^2,4,5^ O estudo PARTNER 2 avaliou TAVI versus SAVR em pacientes de risco intermediário, com TAVI não sendo inferior no composto de mortalidade por todas as causas e acidente vascular cerebral incapacitante.^6^ O SURTAVI avaliou o mesmo perfil, com resultados semelhantes.^7^ Em pacientes de baixo risco, o estudo PARTNER 3 mostrou uma redução no composto de mortalidade por todas as causas, acidente vascular cerebral ou rehospitalização em 1 e 2 anos no grupo TAVI.^8^ Na análise de 5 anos, o composto não foi inferior.^9^ O estudo DEDICATE avaliou 1.414 pacientes de baixo a intermediário risco, com TAVI não sendo inferior no composto de mortalidade por todas as causas e acidente vascular cerebral em 1 ano.^10^ Os resultados apresentados neste estudo são consistentes com a literatura.

Uma subanálise de custo-efetividade do PARTNER 3 mostrou que os materiais para TAVI custam quase US$ 19.000,00 a mais do que para SAVR. No entanto, quando os custos totais de hospitalização foram avaliados, essa diferença caiu para aproximadamente US$ 600,00 e, após 2 anos, os autores concluíram uma economia de US$ 2.000,00 por paciente submetido ao TAVI.^11^ A falta de acompanhamento a longo prazo impede uma avaliação de custo-efetividade, uma limitação destacada pelos próprios autores. Custos adicionais, como transfusão de sangue e hemodiálise, que são mais prováveis de ocorrer após SAVR, não foram detalhados neste estudo.

Procedimentos baseados em cateter têm se tornado cada vez mais comuns em muitas áreas da cardiologia, particularmente em doenças valvares, criando uma subespecialidade em doenças valvares estruturais. Isso começou com a valvoplastia percutânea por balão das valvas mitral e aórtica, seguido pelo TAVI, e agora está consolidando seu uso em outras valvas e próteses valvares. Observamos que pacientes cada vez mais jovens estão sendo submetidos ao TAVI, atingindo até 54% dos pacientes com menos de 65 anos nos Estados Unidos.^12^ Isso ainda não foi abordado por diretrizes nacionais ou internacionais. Novas próteses, estudos demonstrando maior durabilidade e avanços nas técnicas de implantação resultaram em procedimentos cada vez mais seguros.^13^ No entanto, a cirurgia de coração aberto também evoluiu com técnicas menos invasivas, menor tempo de internação hospitalar e menor mortalidade histórica.^14^

A decisão sobre o melhor procedimento deve, portanto, ser considerada com base na idade, comorbidades, risco, viabilidade técnica e compartilhada com o paciente e a família.

Este estudo, embora com limitações, é importante para a análise de dados sobre esses dois procedimentos no cenário brasileiro, particularmente em um hospital público. Acima de tudo, chama a atenção para a relação custo-efetividade e a necessidade de redução de preços de dispositivos e materiais, o que, em última análise, resultará na oferta do melhor tratamento para cada caso no menor tempo possível, um ponto crítico no sistema público de saúde brasileiro.

Em resumo, o TAVI normalmente tem custos iniciais mais altos, mas é compensada por estadias hospitalares mais curtas, levando a custos totais comparáveis ou menores ao longo do tempo em comparação à SAVR, que tem custos iniciais mais baixos, mas hospitalizações mais longas.
