# Supplementary material for: Implicações de Custo e Perspectivas de TAVI versus SAVR no Ambiente Hospitalar
Source: Arq Bras Cardiol. 2025 Nov 27;122(10):e20250669. [Article in Portuguese] doi: 10.36660/abc.20250669 (PMC12671656; doi:10.36660/abc.20250669)
Supplement: Translation

# Translation: Cost Implications and Perspectives of TAVI versus SAVR in the Hospital Setting

**DOI:** 10.36660/abc.20250669i

**Authors:** Renato Paladino Nemoto, Roney Orismar Sampaio

**Affiliations:** 1 São Paulo SP Brazil Instituto do Coração do Hospital das Clínicas da Faculdade de Medicina da Universidade de São Paulo, São Paulo, SP - Brazil

**Keywords:** Costs and Cost Analysis, Transcatheter Aortic Valve Implantation, Transcatheter Aortic Valve Replacement, Therapeutics

The article "Transcatheter Aortic Valve Implantation vs. Aortic Valve Surgery in a Brazilian Public Health System (SUS) Hospital: Periprocedural Outcomes and Costs"^1^ addresses a relevant topic, especially after the inclusion of transcatheter aortic valve implantation (TAVI) in the list of procedures available in the SUS. Patients undergoing TAVI are typically older, with more comorbidities, and are more frail. Conversely, surgical aortic valve replacement (SAVR) in elderly patients can result in increased morbidity and length of hospital stay.^2^

In the study, surgical risk was low in both groups (STS 3.62±2.28 in the TAVI group vs 1.64±0.94 in SAVR, p<0.001), which may demonstrate the limitation of surgical risk stratification with current tools.^3^ Mortality was lower in the TAVI group (2.8% vs. 5.0%), but without reaching statistical significance. The minimally invasive strategy (without general anesthesia, with transthoracic echocardiography monitoring) resulted in a shorter hospital length of stay (2.0 [1.0; 4.0] days in the TAVI group vs. 8.0 [7.0; 12.0] days in the SAVR group, p<0.001). However, the costs were higher than those of SAVR. The high prices of materials (R$ 55,750.90 [52,345.30; 92,286.8] TAVI vs. R$ 22,518.10 [19,130.60; 25,875.10] SAVR, p<0.001) is a significant factor. Late complications, such as a higher rate of hospital infection, including surgical wound infection, are unfavorable to surgery, due to increased hospitalization for use of antimicrobials, hospital human resources, among other costs.

Current national and international guidelines indicate a preference for TAVI in patients at high surgical risk or with significant frailty over SAVR.^2,4,5^ The PARTNER 2 trial evaluated TAVI versus SAVR in intermediate-risk patients, with TAVI being non-inferior in the composite of all-cause mortality and disabling stroke.^6^ The SURTAVI evaluated the same profile, with similar results.^7^ In low-risk patients, the PARTNER 3 trial showed a reduction in the composite of all-cause mortality, stroke, or rehospitalization at 1 and 2 years in the TAVI group.^8^ In the 5-year analysis, the composite was non-inferior.^9^ The DEDICATE trial evaluated 1.414 low- to intermediate-risk patients, with TAVI being non-inferior in the composite of all-cause mortality and stroke at 1 year.^10^ The results presented in this study are consistent with the literature.

A cost-effectiveness subanalysis of PARTNER 3 showed that materials for TAVI cost nearly $19,000.00 more than SAVR. However, when overall hospitalization costs were assessed, this difference dropped to approximately $600.00, and after 2 years, the authors concluded a savings of $2,000.00 per patient undergoing TAVI.^11^ The lack of long-term follow-up precludes a cost-effectiveness assessment, a limitation highlighted by the authors themselves. Additional costs, such as blood transfusion and hemodialysis, which are more likely to occur post-SAVR, were not detailed in this study.

Catheter-based procedures have become increasingly common in many areas of cardiology, particularly in valve disease, creating a subspecialty in structural valve disease. This began with percutaneous balloon valvuloplasty of the mitral and aortic valves, followed by TAVI, and is now consolidating its use in other valves and valve prostheses. We have observed that increasingly younger patients are undergoing TAVI, reaching up to 54% of patients under 65 years old in the United States.^12^ This has not been addressed by national or international guidelines yet. New prostheses, studies demonstrating greater durability, and advances in implantation techniques have resulted in increasingly safe procedures.^13^ However, open-heart surgery has also evolved with less invasive techniques, shorter hospital stay, and lower historical mortality.^14^

The decision regarding the best procedure should therefore be considered based on age, comorbidities, risk, technical feasibility, and shared with the patient and family.

This study, although with limitations, is important for analyzing data on these two procedures in the Brazilian setting, particularly in a public hospital. Above all, it calls for attention to cost-effectiveness and the need to reduce the prices of devices and materials, which will ultimately result in the provision of the best treatment for each case in the shortest possible time, a critical point in the Brazilian public healthcare system.

In summary, TAVI typically has higher initial costs but is offset by shorter hospital stays, leading to comparable or lower total costs over time compared to SAVR, which has lower upfront costs but longer hospitalizations.
